# Prevalence and Predictors of Chronic Postsurgical Pain After Primary Pediatric Scoliosis Surgery: A Tertiary Hospital Study

**DOI:** 10.7759/cureus.108069

**Published:** 2026-04-30

**Authors:** Rayan Muawad, Yasser M Almashari, Joana Mandoorah, Ziyad K Alharbi, Wasan Alenezi, Rayan Almutairi, Mostafa Nagy

**Affiliations:** 1 Pediatric Anesthesia, Ministry of National Guard Health Affairs, Riyadh, SAU; 2 Anesthesia, King Abdulaziz Medical City, Riyadh, SAU; 3 Medicine, College of Medicine, King Saud Bin Abdulaziz University for Health Sciences, Riyadh, SAU; 4 Anesthesia, College of Medicine, King Saud Bin Abdulaziz University for Health Sciences, Riyadh, SAU; 5 Pediatric Anesthesia, King Abdullah Specialized Children's Hospital, Riyadh, SAU

**Keywords:** chronic postoperative pain, functional limitation, pain management, pediatric scoliosis, spinal fusion surgery

## Abstract

Background and aim

Chronic postoperative pain after pediatric scoliosis correction remains a clinically relevant concern despite advancements in perioperative analgesia. This study aimed to determine the prevalence of chronic pain, evaluate functional limitation, and identify demographic and perioperative predictors among pediatric patients undergoing scoliosis correction.

Methodology

This retrospective cross-sectional study was conducted at King Abdullah Specialized Children's Hospital, National Guard Health Affairs (NGHA). All the pediatric patients who underwent scoliosis correction were included using non-probability purposive sampling. Data were extracted from surgical and anesthesia records from 2021 to 2025, with follow-up pain outcomes assessed at three, six, and 12 months for the past four years. All data were analyzed by SPSS version 27 (Armonk, NY: IBM Corp.).

Results

Our study included 99 patients, of whom chronic pain was present in 22 (22.2%) patients at three months, 14 (14.1%) at six months, and eight (8.1%) at 12 months. Overall, 24 (24.2%) patients met criteria for chronic pain. Functional limitations were observed in 27 (27.3%) patients. Most of the patients (76.8%) didn’t report or show any signs of current pain after the coinvestigators contacted their parents by phone, regardless of the time since surgery (whether it had been one year or four years). In multivariable analysis, increasing age was independently associated with lower chronic pain (B = -0.082, p = 0.016), while postoperative morphine infusion was associated with reduced chronic pain (B = -0.497, p = 0.042). Chronic pain and functional limitation were strongly associated with current pain medication use (B = 0.708, p = 0.002; B = 0.308, p = 0.006). Other demographic and perioperative variables were not significant predictors.

Conclusion

Chronic pain affects approximately one-quarter of pediatric scoliosis patients but decreases over time. Younger age and absence of postoperative morphine infusion were associated with higher chronic pain risk. Optimized postoperative pain management may reduce long-term pain burden.

## Introduction

Scoliosis is a well-known vertebral alteration, characterized mainly by lateral curvature of the spine relative to the vertical line and rotation of the vertebra within the curve [1,2]. It consists of structural and non-structural deformities, and to be appraised, there should be at least 10° of spinal angulation on the posterior-anterior radiograph alongside vertebral rotation [1,2]. It can be classified according to its etiology; for example, idiopathic scoliosis is considered to be the most common type, yet as long as the patients do not show any clinical signs of impairment or pain, scoliosis will not be diagnosed properly [3]. Other types of scoliosis, as mentioned earlier, can be identified, yet they are less common, such as congenital, mesenchymal, or scoliosis of a neuromuscular origin [3,4].

Current data show that an incidence of 0.5-1 in every 1000 births worldwide is diagnosed with congenital scoliosis; furthermore, congenital scoliosis constitutes 10% of scoliotic cases. Adolescent idiopathic scoliosis (AIS), defined as a spinal curvature greater than 10° according to Cobb, has a prevalence of 2-3% [3,4]. With this data, we, as pediatricians, can conclude that scoliosis is not a relatively uncommon condition [3,4].

A surgical approach to such a condition is the gold standard for cases with an advanced Cobb angle, and with different types of deformities come different types of approaches [5]. Also, the variety of the level of scoliosis, types, and Cobb angles of curvatures might cause variability between surgeries as an approach and/or recovery [5]. Complications can happen and most of the time can be predicted, especially at high levels of curves, and may lead to an unpleasant experience with the quality of care provided, such as postoperative pain, which is considered to be one of the leading causes of complaints, along with functional restrictions and an extended recovery period [5,6].

Postoperative pain has been heavily discussed on many occasions; for instance, a meta-analysis of spine surgeries concluded that more than 85 thousand patients underwent spine surgeries, 14.97% of whom were experiencing persistent pain [6]. With such complications, psychological deterioration can happen, which O’Donovan et al. concluded in their study of 26 thousand participants of narrative synthesis and 26 meta-analyses, that postoperative complications showed mixed results, some studies correlating the complications with increased anxiety or depression and wound healing, and others denied it [7].

At a young age, such issues should be dealt with promptly to lower the risk of developing chronic pain [8]. This takes us to the goal of our study - establishing the prevalence of chronic pain among pediatric patients who underwent scoliosis surgery and identifying risk factors and the type of management perioperatively that was provided to address such complications.

## Materials and methods

Objectives of the study

The objective of this study was to establish the prevalence of chronic pain among pediatric patients who underwent scoliosis surgeries for the past four years, and to seek risk factors, prevalence of postoperative persistent functional limitations, such as walking, bending, twisting, and the ability to do day-to-day chores, and the best perioperative treatment methods that lowered such complications perioperatively.

Specific objectives

After identifying the prevalence of chronic pain postoperatively, we seek to establish the management done perioperatively to lower such complications and achieve optimal satisfaction among pediatric patients with the care provided in the future.

Study methodology

The coinvestigators collected the data using the chart review method and a data collection sheet to extract the variables. An interview with both parents via phone and video calls after consent was obtained to assess the presence of chronic pain during the first 12 months and current pain in patients with the presence of functional impairment in the first 12 months postoperation. Patients who were less than five years old were assessed using the Children's and Infant's Postoperative Pain Scale (CHIPPS), by asking the parents all the components that were included in the scale and later checking how high the score for each patient was if it met the criteria of postoperative pain or not, with the affirmation of no stimulation to confirm objectivity and rule out any bias. For children who were above five years of age, direct interviews with the presence of both parents were done to assess the presence of chronic postoperative pain and functional impairment in the first 12 months.

Inclusion and exclusion criteria

All pediatric patients who have undergone scoliosis corrections for the first time from 2021 to 2025, regardless of their etiologies, conditions, and sex, were included. Our study excluded patients who were above the age of 17 years, alongside any patients who had only postanesthesia care unit nurses' notes available without actual anesthesia records. The time period was specifically chosen due to the lack of intraoperative anesthesia records before 2021; without them, transferring such data may cause fatal errors, which can introduce bias.

Study area/setting and sampling technique

This retrospective study was conducted at King Abdullah Specialized Children's Hospital (KASCH), National Guard Health Affairs (NGHA) in Riyadh. NGHA is a comprehensive healthcare organization with a wide range of clinical, academic, and research programs, from public health and primary care to tertiary care specialties and sub-specialties, all within three major hospitals in Riyadh. This study was approved by the ethical committee of the KAIMRC-Riyadh. Later on, before the start of the study, a request was made for data to be only of those who were in the NGHA of the capital city of Riyadh, and excluding those who were at both branches of the west and east of NGHA, to ensure the simplicity of the follow-up process, and if needed, an in-person meeting. This study used a non-probability consecutive sampling technique, including all pediatric patients who met the inclusion criteria. This type of sampling suits the type of data collection method and the category of the study.

Statistical analysis plan

The data were analyzed using SPSS version 27 (Armonk, NY: IBM Corp.). Continuous variables were presented as mean ± standard deviation (SD) or median with interquartile range (IQR), while categorical variables were summarized as frequencies and percentages. Associations between categorical variables were assessed using chi-square or Fisher’s exact tests. Differences in continuous variables between groups were evaluated using Mann-Whitney U tests. Multivariable linear regression analysis was performed to identify independent predictors of chronic pain and functional limitations. A p < 0.05 was considered statistically significant.

## Results

Our study included 99 patients who underwent scoliosis correction for assessment of chronic pain postsurgery and persistent functional disability. The majority of the patients were female (70; 70.7%), and most patients were older than 10 years (58; 58.6%). The mean age of the patients was 10.6 (SD: 3.4) years (range: one to 16 years). The estimated blood loss had a median of 200 mL (IQR: 100-300 mL) (Table 1).

**Table 1:** Baseline demographic characteristics of pediatric scoliosis correction patients.

Variables		n (%)
Gender	Female	70 (70.7)
	Male	29 (29.3)
Age (years)	1-5	11 (11.1)
	6-10	30 (30.3)
	>10	58 (58.6)
	Mean ± SD	10.6 (3.4)
	Range	1-16
Estimated blood loss (mL)	Median (IQR)	200 (100-300)

In terms of intraoperative and postoperative management of pain among scoliosis patients, most of the patients received IV acetaminophen (79; 79.8%), whereas non-steroidal anti-inflammatory drugs (NSAIDs) (IV ketorolac) were given to 29 (29.3%) patients. Dexmedetomidine was used in 38 (38.4%) patients, while ketamine was used rarely (8; 8.1%). Nearly all patients underwent general anesthesia alone (97; 98.0%), with a small proportion receiving combined general anesthesia (GA) and spinal anesthesia using preservative-free morphine 3 μg/kg without additional intrathecal agents (2; 2.0%). Intraoperative opioids were given to 97 (98.0%) patients. For postoperative management of pain, as needed (PRN) intravenous morphine boluses were given to 56 (56.6%) patients, IV patient-controlled analgesia (PCA) was used in 30 (30.3%) patients, and morphine was infused in 29 patients according to μg/kg/h in the postanesthesia care unit (PACU) (29.3%). Caudal morphine consisted of preservative-free morphine 30 μg/kg, which was used in 18 (18.2%) patients, whereas epidural analgesia was administered as a continuous infusion of 0.2% ropivacaine without opioid or other adjuncts, which was used rarely (2; 2.0%) (Table 2).

**Table 2:** Intraoperative and postoperative analgesic management. GA: general anesthesia; PCA: patient-controlled analgesia, PRN: as needed; ESP: erector spinae plane nerve block; NSAID: non-steroidal anti-inflammatory drugs; NA: neuraxial anesthesia

Variables		n (%)
Acetaminophen used	No	20 (20.2)
	Yes	79 (79.8)
IV NSAID (ketorolac) used	No	70 (70.7)
	Yes	29 (29.3)
IV dexmedetomidine used	No	61 (61.6)
	Yes	38 (38.4)
IV ketamine used	No	91 (91.9)
	Yes	8 (8.1)
Anesthesia type (GA ± NA)	GA	97 (98.0)
	GA + NA	2 (2.0)
Intraoperative IV opioid used	No	2 (2.0)
	Yes	97 (98.0)
PRN morphine IV boluses (postoperatively)	No	43 (43.4)
	Yes	56 (56.6)
IV PCA used	No	69 (69.7)
	Yes	30 (30.3)
Morphine infusion (μg/kg/h)	No	70 (70.7)
	Yes	29 (29.3)
Caudal morphine (30 μg/kg)	No	81 (81.8)
	Yes	18 (18.2)
Epidural used 0.2% ropivacaine	No	97 (98.0)
	Yes	2 (2.0)
ESP block used	No	99 (100.0)
Postoperative complications	No	99 (100.0)

Table 3 shows the prevalence of postoperative pain over time at each time-phase, and postoperative functional limitation during the first 12 months, and the current use of analgesia (both opioid and non-opioid) among patients (after one to four years according to the time of procedure). At three months postoperatively, 22 (22.2%) patients experienced pain. At six months, the proportion of patients reporting persistent pain decreased to 14 (14.1%). By 12 months, 91 (91.9%) patients remained pain-free, and eight (8.1%) reported pain. Postoperative functional limitation was observed in 27 patients (27.3%), whereas the majority had no limitation (72; 72.7%). Regarding the current usage of pain medication, most of the patients were not taking pain medication at follow-up (80; 80.8%), while there was a smaller proportion that remained on analgesics (both opioid and non-opioid) for many years (one to four years according to the time of procedure) (19; 19.2%). To be safe and as objective as possible, both parents were interviewed to ensure the information provided was accurate, and patients were interviewed if they were five years old or older.

**Table 3:** Prevalence of postoperative pain over time, functional limitation, and current analgesic use (n = 99).

Variables		n (%)
Pain at 3 months	No	77 (77.8)
	Yes	22 (22.2)
Pain at 6 months	No	85 (85.8)
	Yes	14 (14.1)
Pain at 12 months	No	91 (91.9)
	Yes	8 (8.1)
Functional limitation	No	72 (72.7)
	Yes	27 (27.3)
Pain medication use (current usage at the time of contact)	No	80 (80.8)
	Yes	19 (19.2)

Table 4 shows that the association of gender and age with chronic pain was not significant. The estimated blood loss also had no impact on chronic pain (p = 0.641). Preoperative analgesic strategies such as IV acetaminophen, NSAIDs (IV ketorolac), dexmedetomidine, ketamine, regional block type, and intraoperative opioid use were not significantly associated with chronic pain (p > 0.05). Similarly, postoperative modalities showed no significant associations. However, current pain medication (opioid and non-opioid) use was strongly associated with chronic pain, with a higher prevalence among those on medications (11; 57.9%) as compared to those not taking medications (13; 16.3%) (p < 0.001).

**Table 4:** Association between demographic, perioperative factors, and chronic postoperative pain among pediatric scoliosis patients (n = 99). *Chi-square test. **Fisher’s exact test. ***Mann-Whitney U test. PCA: patient-controlled analgesia; PRN: as needed; ESP: erector spinae plane nerve block; NSAID: non-steroidal anti-inflammatory drugs; GA: general anesthesia; NA: neuraxial anesthesia

Variables		Chronic pain, n (%)		Test statistic	Significant values
		No	Yes		
Gender	Female	54 (77.1)	16 (22.9)	χ² = 0.250	0.617*
	Male	21 (72.4)	8 (27.6)		
Age (years)	1-5	8 (72.7)	3 (27.3)	Exact value = 1.170	0.608**
	6-10	21 (70.0)	9 (30.0)		
	> 10	46 (79.3)	12 (20.7)		
Estimated blood loss	Mean (SD)	251.86 (286.76)	287.39 (281.20)	U = 639	0.641***
Intraoperative pain management					
IV acetaminophen used	No	16 (80.0)	4 (20.0)	Exact value = 0.254	0.620**
	Yes	59 (74.7)	20 (25.3)		
IV NSAID (ketorolac) used	No	55 (78.6)	15 (21.4)	χ² = 1.030	0.310*
	Yes	20 (69.0)	9 (31.0)		
IV dexmedetomidine used	No	43 (70.5)	18 (29.5)	χ² = 2.399	0.121*
	Yes	32 (84.2)	6 (15.8)		
IV ketamine used	No	68 (74.7)	23 (25.3)	Exact value = 0.143	0.419**
	Yes	7 (87.5)	1 (12.5)		
Anesthesia type	GA	73 (75.3)	24 (24.7)	Exact value = 0.737	1.000**
	GA + spinal	2 (100)	0 (0)		
Intraoperative IV opioid	No	2 (100)	0 (0)	χ² = 0.653	0.419*
	Yes	73 (75.3)	24 (24.7)		
Postoperative pain management					
IV PRN morphine	No	33 (76.7)	10 (23.3)	χ² = 0.040	0.841*
	Yes	42 (75.0)	14 (25.0)		
IV PCA used	No	51 (73.9)	18 (26.1)	χ² = 0.422	0.516*
	Yes	24 (80.0)	6 (20.0)		
Morphine infusion (μg/kg/h)	No	50 (71.4)	20 (28.6)	Exact value = 1.700	0.133**
	Yes	25 (86.2)	4 (13.8)		
Caudal morphine (30 μg/kg)	No	60 (74.1)	21 (25.9)	χ² = 0.687	0.407*
	Yes	15 (83.3)	3 (16.7)		
Epidural (ropivacaine 0.2%)	No	73 (75.3)	24 (24.7)	Exact value = 0.000	1.000**
	Yes	2 (100)	0 (0)		
Current pain medication use	No	67 (83.8)	13 (16.3)	χ² = 14.499	<0.001*
	Yes	8 (42.1)	11 (57.9)		

Table 5 shows a multivariable regression analysis. There are only three variables that were significantly associated with chronic pain after multivariable adjustment. The increase in age was independently associated with lower chronic pain (B = -0.082, p = 0.016). Postoperative morphine infusion according to μg/kg/h without demand was also significantly associated with reduced chronic pain (B = -0.497, p = 0.042). In contrast, current pain medication (opioid and non-opioid) use showed a strong positive association with chronic pain (B = 0.708, p = 0.002), suggesting that patients with greater chronic pain are more likely to require ongoing analgesic medication. All other sociodemographic, intraoperative, and postoperative variables were not statistically significant predictors (p > 0.05).

**Table 5:** Multivariable linear regression analysis for chronic pain. SE: standard error; LL: lower limit; UL: upper limit; PCA: patient-controlled analgesia; PRN: as needed; ESP: erector spinae plane nerve block; NSAID: non-steroidal anti-inflammatory drugs; GA: general anesthesia; NA: neuraxial anesthesia

Variables	B (SE)	Beta	t	p-Value	95% CI (LL to UL)
Sociodemographic parameters					
Gender	0.396 (0.233)	0.181	1.697	0.094	-0.068 to 0.860
Age	-0.082 (0.033)	-0.274	-2.468	0.016	-0.148 to -0.016
Estimated blood loss	0.001 (0.000)	0.165	1.485	0.142	0.000 to 0.001
Intraoperative management					
IV acetaminophen	-0.018 (0.240)	-0.008	-0.075	0.941	-0.494 to 0.458
IV NSAID (ketorolac)	0.226 (0.207)	0.116	1.095	0.276	-0.184 to 0.637
IV dexmedetomidine	-0.155 (0.194)	-0.085	-0.798	0.427	-0.541 to 0.231
IV ketamine	-0.063 (0.345)	-0.019	-0.183	0.855	-0.748 to 0.621
Anesthesia type (GA ± NA)	-0.116 (0.547)	-0.023	-0.212	0.832	-1.202 to 0.970
Intraoperative opioid	0.486 (0.655)	0.077	0.741	0.460	-0.816 to 1.787
Postoperative management					
IV PRN morphine	-0.264 (0.222)	-0.147	-1.187	0.238	-0.705 to 0.178
IV PCA	-0.327 (0.229)	-0.169	-1.432	0.156	-0.781 to 0.127
Morphine infusion (μg/kg/h)	-0.497 (0.241)	-0.254	-2.060	0.042	-0.976 to -0.018
Caudal morphine (30 μg/kg)	-0.106 (0.230)	-0.046	-0.459	0.648	-0.563 to 0.352
Epidural (ropivacaine 0.2%)	0.427 (0.708)	0.068	0.604	0.547	-0.978 to 1.833
Current pain medication use	0.708 (0.219)	0.313	3.230	0.002	0.273 to 1.143

Table 6 shows that there was no significant association between functional limitation and gender, age group, or estimated blood loss (p > 0.05). Intraoperative analgesic modalities, which included IV acetaminophen, IV NSAIDs (ketorolac), dexmedetomidine, ketamine, anesthesia type, and intraoperative opioid use, were not significantly associated (p > 0.05). Postoperative variables were similarly non-significant, although IV PRN morphine showed borderline significance (p = 0.052). Only current pain medication (opioid and non-opioid) use was significantly associated with higher limitation among users (10; 52.6%) versus non-users (17; 21.3%) (p = 0.006).

**Table 6:** Association between clinical factors and functional limitation. *Chi-square test. **Fisher’s exact test. ***Mann-Whitney U test. PCA: patient-controlled analgesia; PRN: as needed; ESP: erector spinae plane nerve block; NSAID: non-steroidal anti-inflammatory drugs; GA: general anesthesia; NA: neuraxial anesthesia

Variables		Functional limitations, n (%)		Test statistic	Significant values
		No	Yes		
Gender	Female	53 (75.7)	17 (24.3)	χ² = 1.075	0.300*
	Male	19 (65.5)	10 (34.5)		
Age (years)	1-5	9 (81.8)	2 (18.2)	Exact value = 0.944	0.575**
	6-10	23 (76.7)	7 (23.3)		
	>10	40 (69.0)	18 (31.0)		
Estimated blood loss	Mean (SD)	249.82 (305.10)	231.85 (231.83)	U = 564	0.641***
Intraoperative management					
IV acetaminophen	No	17 (85.0)	3 (15.0)	Exact value = 1.884	0.168**
	Yes	55 (69.6)	24 (30.4)		
IV NSAID (ketorolac)	No	51 (72.9)	19 (27.1)	χ² = 0.002	0.964*
	Yes	21 (72.4)	8 (27.6)		
IV dexmedetomidine	No	43 (70.5)	18 (29.5)	χ² = 0.400	0.527*
	Yes	29 (76.3)	9 (23.7)		
IV ketamine	No	66 (72.5)	25 (27.5)	Exact value = 0.022	0.880**
	Yes	6 (75.0)	2 (25.0)		
Anesthesia type (GA ± NA)	GA	70 (72.2)	27 (27.8)	Exact value = 0.831	0.570**
	GA + spinal	3 (100)	0 (0)		
Intraoperative IV opioid	No	1 (50.0)	1 (50.0)	Exact value = 0.526	0.466**
	Yes	71 (73.2)	26 (26.8)		
Postoperative management					
IV PRN morphine	No	27 (62.8)	16 (37.2)	χ² = 3.784	0.052*
	Yes	45 (80.4)	11 (19.6)		
IV PCA used	No	52 (75.4)	17 (24.6)	χ² = 0.797	0.372*
	Yes	20 (66.7)	10 (33.3)		
Morphine infusion (μg/kg/h)	No	51 (72.9)	19 (27.1)	χ² = 0.002	0.964*
	Yes	21 (72.4)	8 (27.6)		
Caudal morphine (30 μg/kg)	No	56 (69.1)	25 (30.9)	Exact value = 2.868	0.089**
	Yes	16 (88.9)	2 (11.1)		
Epidural (ropivacaine 0.2)	No	70 (72.2)	27 (27.8)	Exact value = 0.758	0.382**
	Yes	2 (100)	0 (0)		
Pain medication use (current)	No	63 (78.8)	17 (21.3)	χ² = 7.623	0.006*
	Yes	9 (47.4)	10 (52.6)		

Table 7 shows the multivariable linear regression analysis for functional limitation. After adjusting for sociodemographic, intraoperative, and postoperative variables, functional disability emerged as the strongest predictor of current pain-medication use due to chronic pain. Patients with greater functional limitation were significantly more likely to report using pain medication (opioid and non-opioid) due to chronic pain (B = 0.308, p = 0.006). Other variables, including gender, age, and estimated blood loss, were not significant predictors in the adjusted model. Similarly, preoperative and postoperative analgesic strategies showed no independent association with pain medication use.

**Table 7:** Multivariable linear regression analysis for functional limitation. SE: standard error; LL: lower limit; UL: upper limit; PCA: patient-controlled analgesia; PRN: as needed; ESP: erector spinae plane nerve block; NSAID: non-steroidal anti-inflammatory drugs; GA: general anesthesia; NA: neuraxial anesthesia

Variables	B (SE)	Beta	t	p-Value	95% CI (LL to UL)
Sociodemographic parameters					
Gender	0.099 (0.119)	0.094	0.830	0.409	-0.138 to 0.336
Age (years)	0.001 (0.017)	0.007	0.058	0.954	-0.033 to 0.035
Estimated blood loss	0.00009 (0.000)	0.057	0.481	0.632	0
Intraoperative management					
IV acetaminophen	0.174 (0.120)	0.157	1.458	0.148	-0.063 to 0.412
IV NSAID (ketorolac)	-0.024 (0.103)	-0.025	-0.236	0.814	-0.229 to 0.180
IV dexmedetomidine	-0.060 (0.097)	-0.065	-0.618	0.538	-0.253 to 0.133
IV ketamine	-0.042 (0.172)	-0.026	-0.245	0.807	-0.383 to 0.299
Anesthesia type (GA ± NA)	-0.123 (0.273)	-0.048	-0.451	0.653	-0.664 to 0.418
Intraoperative IV opioid	-0.204 (0.327)	-0.064	-0.623	0.535	-0.853 to 0.445
Postoperative management					
IV PRN morphine	-0.181 (0.112)	-0.201	-1.619	0.109	-0.403 to 0.041
IV PCA	0.053 (0.115)	0.054	0.459	0.648	-0.176 to 0.281
Morphine infusion (μg/kg/h)	-0.057 (0.121)	-0.058	-0.469	0.640	-0.298 to 0.184
Caudal morphine (30 μg/kg)	-0.163 (0.116)	-0.141	-1.406	0.163	-0.393 to 0.067
Epidural (ropivacaine 0.2%)	-0.081 (0.356)	-0.026	-0.228	0.820	-0.789 to 0.626
Pain medication use	0.308 (0.110)	0.272	2.791	0.006	0.089 to 0.527

## Discussion

Chronic pain after scoliosis surgery remains a significant concern that affects about 15% of patients long-term and potentially impairs recovery [6]. There are several psychological factors, including anxiety and depression, which further worsen pain outcomes and functional recovery after surgery [7]. This study evaluated chronic postoperative pain and functional limitations among pediatric patients undergoing scoliosis correction. Notably, chronic pain was present in approximately one-quarter of patients at three months (22; 22.2%), which reduced to 14 (14.1%) at six months and eight (8.1%) at 12 months. Overall, 24.2% of patients met the criteria for chronic pain at follow-up, while 27 (27.3%) experienced functional limitations consisting of one or more of the following: walking, bending, and twisting. Encouragingly, most patients reported no current pain (76.8%), indicating that long-term outcomes after scoliosis correction are generally favorable and align with the study by Barile et al. [8]. However, there is still a subset of patients who continue to experience persistent symptoms.

The observed chronic pain prevalence aligned with the previously reported rates of persistent postoperative pain after spinal deformity surgery, which ranged from 12% to 42% in pediatric and adolescent populations [9]. Moreover, prior literature on adolescent idiopathic scoliosis (AIS) has shown that while surgical correction improves deformity and quality of life, a small subset of patients report ongoing back pain months to years after surgery [10]. Multifactorial mechanisms have been proposed, which include central sensitization, surgical tissue trauma, neuropathic components, and psychosocial factors. Our findings also reinforce that chronic pain remains a relevant postoperative concern despite advances in perioperative analgesia.

Importantly, this study shows that age demonstrated an independent inverse association with chronic pain. Younger patients were more likely to experience persistent pain. This finding may reflect differences in perception of pain, mechanisms to cope with pain, or neurodevelopmental pain processing. There are some pediatric pain studies, such as the study by Hoehn et al., which suggested that younger children may have less mature descending inhibitory pathways, which potentially increase vulnerability to persistent pain states [11]. Additionally, younger patients may face greater challenges in communicating pain or adapting functionally after major spine surgery. While many AIS cohorts predominantly include adolescents, our inclusion of younger age groups may explain the observed age-related effect.

Furthermore, the utilization of postoperative morphine infusion according to μg/kg/h in PACU and the admission ward was independently associated with lower chronic pain. This suggests that adequate early postoperative analgesia may play a protective role against pain chronification. Similarly, Matamala et al. show that promptly and adequately treating acute postoperative pain can reduce the risk that it will transition into chronic postoperative pain [12]. The concept that aggressive acute pain control reduces the risk of persistent pain is supported in broader surgical literature. There are also previous studies in adult thoracic and orthopedic surgery that demonstrate poorly controlled acute postoperative pain increases the likelihood of chronic pain development [13]. Effective opioid-based multimodal analgesia during the acute phase may attenuate central sensitization and limit long-term nociceptive amplification. Although causality cannot be definitively established in our design, the association supports the principle of optimized acute pain management.

The use of current opioid and non-opioid pain medication was strongly associated with both chronic pain and functional limitations. All the patients who actively use analgesics were more likely to report persistent pain and reduced function. This showed that the use of medication reflects the ongoing burden of symptoms rather than directly causing poorer outcomes. These findings aligned with the musculoskeletal pain literature, in which persistent analgesic use correlates with higher pain severity and disability. There is a study by Samuelsen et al. who reported that the prevalence of pain medication usage was higher (10%) among individuals with chronic pain as compared to the general population (4%) [14]. Therefore, use of the medication at follow-up may serve as a clinical indicator of the unresolved pain rather than a modifiable risk factor.

Notably, most intraoperative medications such as acetaminophen, NSAIDs, dexmedetomidine, ketamine, the type of anesthesia, and intraoperative opioids were not significantly associated with chronic pain or functional limitation. However, a previous study by Mercanoglu et al. showed that agents such as dexmedetomidine and ketamine can reduce the requirements of acute opioids, but their impact on preventing long-term chronic pain is still debatable [15]. Similarly, regional and multimodal analgesic strategies also improve early recovery but do not consistently prevent chronic postoperative pain.

These findings of this study further show that estimated blood loss was not associated with chronic pain or functional limitation. Although greater surgical magnitude and tissue trauma could theoretically increase the risk of long-term pain. However, our findings showed that perioperative physiological stress, as reflected by blood loss, may not independently predict chronic outcomes in this population.

Notably, one of the important findings is that functional disability is a predictor variable of both chronic pain and the use of pain medications. However, most of the demographic and perioperative variables were not independently associated, which reinforced that persistent pain itself likely drives long-term functional impairment. The borderline association of PRN morphine (when asked for) and the trend with caudal morphine likely reflected the underlying pain severity rather than direct causation.

Implications and future direction

Effective management of postoperative pain and long-term follow-up in pediatric scoliosis patients is very important. Early identification of patients who are at risk for persistent pain may help to guide targeted interventions and rehabilitation strategies to improve functional outcomes. The association between younger age and chronic pain showed that age-specific pain management approaches may be beneficial. The studies in future research should focus on prospective multicenter studies with larger sample sizes to better understand predictors of chronic pain.

Limitations

There are several limitations of this study that should be acknowledged. The single-center design of this study, with a relatively small sample size, limited the generalizability of the findings. The retrospective nature of the study may also introduce selection and information bias. Additionally, the psychological factors, preoperative levels of pain, and socioeconomic variables were not assessed, which may influence the chronic pain outcomes. The loss to follow-up at later points in time could also affect the accuracy of long-term pain prevalence estimates.

## Conclusions

This study shows that chronic pain persists in 24 (24.2%) of 99 patients following scoliosis correction, but tends to decrease over time. Most of the patients reported no pain and no functional limitations at follow-up. Younger age, being less than 10 years, and the absence of postoperative morphine infusion were associated with higher chronic pain, while the use of current pain medication (opioid and non-opioid) was strongly associated with both chronic pain and functional limitation. Most intraoperative and postoperative analgesic strategies were not associated with chronicity of pain.
